# Decision-making in burst fractures of the thoracolumbar and lumbar spine

**DOI:** 10.4103/0019-5413.36986

**Published:** 2007

**Authors:** Robert F Heary, Sanjeev Kumar

**Affiliations:** Department of Neurological Surgery, University of Medicine and Dentistry of New Jersey, Newark, New Jersey, USA

**Keywords:** Burst fracture, lumbar fracture, thoracolumbar fracture

## Abstract

The most common site of injury to the spine is the thoracolumbar junction which is the mechanical transition junction between the rigid thoracic and the more flexible lumbar spine. The lumbar spine is another site which is more prone to injury. Absence of stabilizing articulations with the ribs, lordotic posture and more sagitally oriented facet joints are the most obvious explanations. Burst fractures of the spine account for 14% of all spinal injuries. Though common, thoracolumbar and lumbar burst fractures present a number of important treatment challenges. There has been substantial controversy related to the indications for nonoperative or operative management of these fractures. Disagreement also exists regarding the choice of the surgical approach. A large number of thoracolumbar and lumbar fractures can be treated conservatively while some fractures require surgery. Selecting an appropriate surgical option requires an in-depth understanding of the different methods of decompression, stabilization and/or fusion. Anterior surgery has the advantage of the greatest degree of canal decompression and offers the benefit of limiting the number of motion segments fused. These advantages come at the added cost of increased time for the surgery and the related morbidity of the surgical approach. Posterior surgery enjoys the advantage of being more familiar to the operating surgeons and can be an effective approach. However, the limitations of this approach include inadequate decompression, recurrence of the deformity and implant failure. Though many of the principles are the same, the treatment of low lumbar burst fractures requires some additional consideration due to the difficulty of approaching this region anteriorly. Avoiding complications of these surgeries are another important aspect and can be achieved by following an algorithmic approach to patient assessment, proper radiological examination and precision in decision-making regarding management. A detailed understanding of the mechanism of injury and their unique biomechanical propensities following various forms of treatment can help the spinal surgeon manage such patients effectively and prevent devastating complications.

Each year, there are approximately 5 million new vertebral fractures worldwide.^1^ In the United States of America, 72.5% of all spinal fractures involve the thoracic or lumbar spines.^2^ The thoracolumbar junction, due to its mechanical transition zone and the lumbar spine, due to its absence of stabilizing articulations with the ribs, lordotic posture and more sagitally oriented facet joints, are reasonable explanations for their involvement in spinal injuries.^3^

In 1963, Holdsworth described burst fractures.^4^ The incidence of burst fractures is maximum at the thoracolumbar junction and occurs frequently in high-energy traumas which are most commonly associated with falls and traffic accidents.^5^ The treatment of thoracolumbar and lumbar burst fractures has remained controversial due to many different options of nonoperative or operative management.^6^

Different factors which play vital roles in the management of such patients include the neurological status, the number of segments involved, the type of the injury and other factors such as the age of the patient, the quality of bone and associated comorbidities. An algorithmic approach is vital for the initial patient assessment, radiological workup and decision-making for ultimate management.

## PRELIMINARY EVALUATION

Patients must be immobilized at first. Airway, breathing and circulation (ABC) must be stabilized before proceeding for the neurological examination. It is not uncommon to have progression of a neurological deficit. Hence, recording of the baseline neurological status and serial assessments thereafter are vital. These should include assessing sensation in each dermatome and at least five muscles or movements should be graded for each extremity. Deep tendon reflexes should also be examined.^7^ A rectal examination is useful in assessing the anal sphincter tone and perianal sensations. Although spinal shock does not usually last for more than 24 h, it may last for days to weeks. Return of the anal wink reflex usually indicates the end of spinal shock. A progressive neurological deficit is a widely accepted indication for urgent surgical intervention. The possibility of a spinal fracture cannot be ruled out with a normal neurological examination as the majority of thoracolumbar injuries do not have associated neurological deficits.^2^

As the spinal cord can variably terminate between T11 to L2, a variety of neurological deficits can result from burst fractures of the thoracolumbar and lumbar spines ranging from frank spinal cord injury to a cauda equine syndrome.

## RADIOLOGICAL EXAMINATION

**Figure 1.1:**
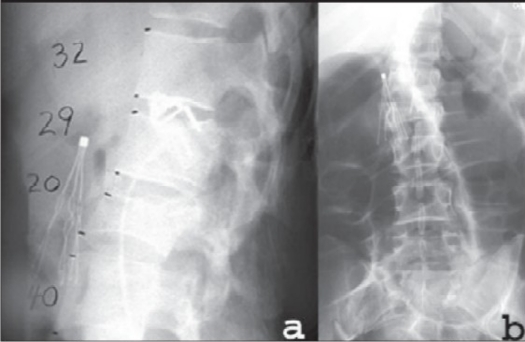
Lateral plain film radiograph (a) demonstrates a 17% loss of height at L1 and a 50% loss of height at L2. AP plain film (b) radiograph demonstrates a focal translation at the level of the L1-L2 subluxation causing a coronal plane deformity. An inferior vena caval filter is visualized

**Figure 1.2:**
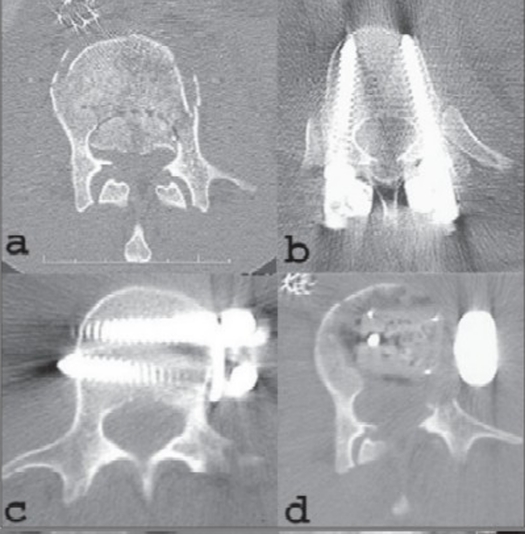
Axial CT scan (a) image at the level of the L2 pedicles demonstrates a 70% compromise of the spinal canal area by a large retropulsed fragment of the vertebra. Of note, the L1 vertebra had a 55% loss of spinal canal area. The patient was operated for -AP spinal reconstruction: Anterior surgery includes corpectomies of L1 and L2, placement of a stackable carbon fiber cage filled with autograft bone anteriorly from T12 - L3 and stabilization with a Kaneda screw-rod construct. Posteriorly the stabilization with bilateral pedicle screws at T11 and L4, bilateral hooks at T12 and L3, two rods with two crossconnectors and generous amounts of autologous iliac crest bone graft was done. (b) Axial CT scan at the level of the T11 pedicles demonstrates well-positioned pedicle screws which approach the far bony cortex of the T11 vertebra. Axial CT scan (c) at the level of the L3 pedicles demonstrates the inferior Kaneda screws which are placed across the vertebra to achieve bicortical fixation. Axial CT scan (d) through the L2 level which shows the pedicle-to-pedicle decompression of the spinal canal with the carbon fiber cage filled with autograft

Obtaining an anteroposterior (AP) and lateral plain radiograph of the suspected involved segment is the standard practice for the initial assessment of the patient [Figure 1.1.] However, plain radiographs sometimes fail to demonstrate some of the important aspects of the spinal fractures. Recently, computerized tomography (CT) scanning is being increasingly utilized in conjunction with plain radiographs. A CT scan provides more diagnostic information than plain radiographs regarding the extent of bony injury.^8^ Another advantage of the CT scan is its ability to better assess the degree of canal compromise [Figure 1.2.] James *et al*., (2005), documented that laminar and articular process fractures are typically missed on plain radiographs and are best visualized on axial CT scans.^9^ However, it is our practice to obtain AP and lateral plain films of the region if an injury is suspected as these radiographs are useful for preoperative planning and postoperative followups. In addition, subtle changes in the soft tissues or between the posterior elements can alert the physician to areas requiring further examination. Kyphotic and translation injuries can be visualized on sagittal and coronal reconstructions. Vertebral body height, disc spaces, inter-pedicular distances and inter-spinous process intervals must be noted and compared between the injured and the non-injured levels. However, CT scans have a limited role in visualizing soft-tissue injuries which include disc herniations, epidural or subdural hematomas, ligamentous injuries and spinal cord parenchymal injury.^3^

Magnetic resonance (MR) imaging has the ability to visualize the soft-tissue components of spinal injuries.^3^ Its utility for the thoracolumbar junction is important due to the variable location of the conus medullaris in the adult population.^9^ Biomedical implants, such as cardiac pacemakers and aneurysm clips, are contraindications for MR imaging. We reserve MR imaging for patients with a neurological deficit or in whom the integrity of the posterior ligamentous complex is questionable. For such patients, the short tau inversion recovery (STIR) sequence is particularly valuable for detecting acutely injured soft tissues. In patients who cannot undergo an MR imaging study, a myelogram followed by a post-myelogram CT scan is a reasonable alternative imaging study.

## CLASSIFICATION

Many different classification systems have been proposed for thoracolumbar and lumbar burst fractures. Holdsworth proposed a two-column model of spinal stability by separating the spine into an anterior weight-bearing column of the vertebral body and a posterior tension-bearing column of the posterior ligamentous complex (PLC). He termed burst fractures as unstable if the PLC was disrupted.^4^ Denis (1983) described a three-column classification of spinal fractures. He proposed that injury to the middle column i.e. the posterior portion of the vertebral body, posterior longitudinal ligament and posterior disc was sufficient to create instability.^10^ He also classified unstable fractures into three types: mechanical (1^st^ degree), neurological (2^nd^ degree) or combined mechanical/neurological (3^rd^ degree). In 1994, McAfee *et al.* proposed another classification and treatment scheme. He classified the injuries based on how the middle column failed, with burst fractures exhibiting middle column failure in compression. He also distinguished between burst fractures with and without PLC disruption.^11^ A burst fracture with PLC disruption is considered to be unstable. It is widely accepted that the posterior ligaments have probably failed if there is greater than 30° of kyphosis and/or 50% of vertebral body height loss on plain radiographs.

McCormack *et al.,* also in 1994, proposed another classification which was based on the load-sharing basis. They specifically designed their classification based on the relevance to thoracolumbar burst fractures. They used a point-based system which grades the amount of vertebral body comminution, displacement of fracture fragments and the degree of kyphosis.^12^ The aim of this load-sharing system was to predict the failure of short-segment posterior fixation for a burst fracture as it suggests that injuries with high scores should undergo supplemental anterior column support.

## MECHANISM OF INJURY

Due to gravity in the upright posture, an axial load is exerted on the vertebral column and the body's center of gravity passes anterior to the thoracic spine, through the thoracolumbar junction, posterior to the lumbar spine and through the sacral promontory. With sudden acceleration or deceleration, an increase in axial loads, with or without flexion or extension, can lead various components of the vertebral column to fail. Multiple fracture lines propagate due to axial loading of the vertebral body in burst fractures which can lead to discontinuity of the posterior vertebral body and the adjacent pedicles. The explosive nature of a burst fracture can lead to variable degrees of vertebral body retropulsion into the canal as well.

The comprehensive classification system by Magerl, which has further been modified by the AO group, has classified burst fractures. Type A injuries are axial compression injuries. Type B injuries are distraction injuries including flexion-distraction injuries. Type C injuries are unstable three-column injuries with rotation in the anteroposterior projection. According to this classification, all burst fractures are compression fractures and may be stable or unstable. Hence, it is important to differentiate Type A-3 fractures from Type C-1 fractures (where all three columns fail leading to a higher degree of instability). Although these classification systems provide some guidelines to the many varieties of thoracolumbar burst fractures, different combinations do exist, requiring careful assessment to define the mechanical failure that has occurred at the site of the injury.

The most unstable variant of the burst fracture is where significant kyphosis (more than 30°) is present, with or without 50% of vertebral body height loss, on plain radiographs. These injuries are typically associated with posterior ligamentous injury or horizontal posterior element fracture. This injury type, is clinically suspected by marked posterior tenderness, bruising or a palpable gap at the interspinous level. In unstable burst fractures, the anterior and middle columns fail under axial compression and the posterior column fails due to tension.

## PRINCIPLES OF SURGICAL TREATMENT

The three basic components of surgical treatment of thoracolumbar and lumbar burst fractures include neural decompression, stabilization and fusion. A coherent and logical rationale must be followed in order to achieve the desired results. However, it is always advisable to make individualized decisions in every case.

## NEUROLOGICAL DECOMPRESSION

The need for neural decompression can broadly be divided into two groups of patients- one with neurological deficit and the other without it.

### 1. Patients with neurological deficit

Surgery is usually considered as the primary line of treatment for these patients with the goal of achieving decompression of the neural elements. It has been documented in the past that neurological recovery following decompression has a better prospect than the recovery seen after conservative treatment.^6^ The methods of decompression can vary depending on the personal choice and experience of the operating surgeon. However, it has been reported that greater neurological improvement can be achieved following anterior decompression as compared to posterior or posterolateral.^13^ Kaneda *et al.*, (1984), documented that anterior decompression results in a maximum canal decompression.^14^ Bradford *et al.*, reported an average 25.9% of residual canal compromise following posterior surgery compared to less than 1% after anterior decompression.^15^ Belanger *et al*. 2005, reported that even in cases of long standing compression, anterior decompression can result in modest improvements in neurological function.^16^ In our practice, we usually perform posterior surgery for cases with complete motor-sensory American Spinal Injury Association (ASIA) class A spinal cord injuries (SCI). The extent of instrumentation is usually two or three levels above and two levels below.

Cases with partial neural deficits are ideal candidates for anterior decompression as they have the greatest chance for neurological recovery. Posterior decompression alone using laminectomy does not result in an effective decompression of the neural elements.^6^ However, it is not the absolute indication for the surgery. Patients with progressively increasing neurological deficit may be considered as an absolute indication for the surgery. In patients with entrapped nerve roots, laminectomy is commonly performed in addition to an anterior decompression to release the nerve roots.^17^ For cases with incomplete neurological injuries (ASIA classes B-D) with significant ventral bony compression (50% or more on axial CT scan), no motor deficit with only bowel and bladder dysfunction or significant kyphotic deformity, we prefer anterior surgery. However, in the majority of cases, either an anterior or a posterior approach is reasonable.

**Figure 1.3:**
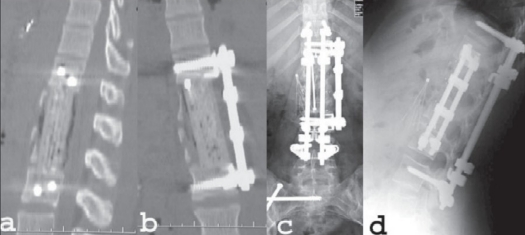
Sagittal reconstruction of a CT scan (a) of the same patient as fig 1.1 & 1.2 demonstrating good positioning of the stackable carbon fiber cage with complete decompression of the spinal canal. Kaneda screws are seen at T12 and L3. Coronal reconstruction of a CT (b) scan demonstrating good positioning of the stackable carbon fiber cage and the Kaneda screw-rod fixation device. AP plain film radiograph (c) obtained two years after surgery demonstrates solid fusion and excellent alignment. Lateral plain film radiograph (d) at two years postoperatively demonstrates preservation of sagittal alignment. At five years post-injury, the patient was neurologically intact with normal bowel and bladder function. She was gainfully employed and despite a solid T11-L4 fusion, she was able to forward bend and touch her toes with her knees fully extended. She required no pain medications

The choice of approach primarily depends on the experience and preference of the operating surgeon and his/her results. In our practice, we have a multidisciplinary team which enables us to safely perform ventral surgery in the acute post-injury period. In our experience, blood loss, neurological outcomes and overall management morbidity and mortality have been very low from either approach. The use of postoperative CT scans has demonstrated very good canal decompression and improvement of sagittal plane alignment when anterior surgery is performed for significant burst fractures with either significant canal compromise or significant kyphosis at the level of the injury [Figure 1.3]. The most important factor determining the operative approach is the individual surgeon's own outcomes which lead to the best neurological and functional outcomes. In the overwhelming majority of patients, successful decompression and stabilization/fusion can be accomplished by either approach.

For cases with severe three-column instability and preserved neurological function we have found it necessary to perform both anterior and posterior surgery (AP) [Figure 1.1–1.3.] In medically stable, younger patients, we routinely perform the AP surgeries under a single anesthetic session. For less stable patients, staged surgeries with two separate operations are occasionally necessary.

Because of the more fluid nature of the dural sac of the cauda equina, lumbar burst fractures with neurological deficits have been reported to be at a higher risk. If this injury is left untreated, it can lead to neural element herniations, entrapment, epidural or subdural hematoma, cerebrospinal fluid leak and even pseudomeningocele.^18^

Another approach for anterior decompression is the lateral extracavitary approach. This approach has the advantage of providing access to both the anterior and posterior elements using a single dorsal incision. A hockey stick-shaped incision can provide access to the area beneath the paraspinal muscles to allow for lateral visualization of the thecal sac. Ventral dissection exposes the lateral border of the injured vertebral body for fragment removal and reconstruction. However, this approach is technically demanding with a very high rate of complications. Resnick *et al*. documented a complication rate as high as 55% with this approach.^19^ We usually recommend this approach in older patients, morbidly obese patients or medically unstable patients who may be unable to withstand a thoracotomy for pulmonary reasons.

Neurological deficits have been reported in approximately 50% of cases with lumbar burst fractures.^10^ Treatment of low lumbar (L3 to L5) burst fractures is technically different compared to that of thoracolumbar (T10 to L2) fractures. Corpectomy via an anterior approach is usually possible for lower lumbar fractures but becomes increasingly difficult with each lower level. Low lumbar surgeries performed anteriorly can be difficult at times due to the iliopsoas muscle which hinders both the decompression as well the fusion. Aggressive dissection of this muscle can lead to postoperative hip flexor weakness. It is our preference to operate via a posterior approach when the construct extends to and beyond the L4 level.

#### Timing of surgery

The optimal timing of the decompression is another critical aspect of the surgery. A progressive neurological deficit is one of the few indications for immediate surgery to treat a thoracolumbar or lumbar burst fracture. However, most of the studies in this regard have demonstrated no direct correlation between the timing of surgery and the amount of neurological recovery.^6^,^20^ Mirza *et al. (1999)*, in their retrospective study, found improved neurological recovery with surgery within 72 h as compared to surgery within 10 to 14 days.^21^ Oftentimes, early surgery can be more difficult than late due to local soft-tissue conditions, increased operative blood loss and associated visceral or skeletal injuries especially via an anterior approach. The operative trauma from decompression of an acutely edematous spinal cord can itself lead to further neurological trauma.^6^

Neurological deficit is primarily determined by the degree of trauma which occurred to the neural elements at the time of impact. Cauda equina injuries are less likely to have complete neurological deficit, primarily due to its anatomy, when compared to conus medullaris or spinal cord injuries.^13^ A complete injury has no motor, sensory or bladder/bowel function distal to the fractured level when spinal shock has resolved, which usually occurs by 48 h.^6^,^20^ The role of serial examinations over a period of at least two days comes into play to determine this. Decompression of such cases, with a complete injury, is unlikely to result in neurological recovery;^6^ however, the benefits of surgical stabilization can facilitate rehabilitation in these situations. Controversy still remains regarding decompressing the neural elements at the caudal aspect of the spinal cord which may decrease the development of post-traumatic syringomyelia.

### 2. Patients without neurological deficit

Hu *et al.* (1996), reported that the majority of cases with thoracolumbar and lumbar burst fractures are neurologically intact.^2^ Cases with no neurological deficit may be managed conservatively. Cases with a mechanically unstable burst fracture, defined by a disrupted PLC, without any neurological deficit, should be considered as unstable as they are at high risk for neurological decline without surgical stabilization.

It is important to determine the integrity of the PLC in this group of patients. It is widely accepted that the posterior ligaments have probably failed if there is greater than 30° of kyphosis and/or 50% of vertebral body height loss. However, different studies in the past have reported that even with these positive signs many patients can be successfully treated conservatively.^22^,^23^ Recently, MR imaging has been used to determine the continuity of the PLC.

Once PLC disruption is confirmed, stabilization and fusion should be considered for neurologically intact patients. The presence of substantial canal compromise is indication enough for decompression as well. Some surgeons consider an indirect decompression, by distracting a posterior pedicle-screw construct, safer than anterior decompression. However, a risk of neural injury is there during manipulation and removal of retropulsed bone fragments. Furthermore, posterior distraction maneuvers can lead to relative kyphosis with worsening of the global sagittal spinal balance. Wood *et al.* (2005), suggested removal of sufficient vertebral body to allow insertion of a strut without entering the spinal canal.^24^ There is some concern regarding leaving bone fragments in the canal. However, there is hardly any clinical data to support this concern. Cantor *et al*., reported that resorption of the fragments occurs over time with both braces and posterior stabilization alone.^25^ It is our practice to perform a pedicle-to-pedicle decompression of all bone fragments when anterior surgery is performed.

Thoracolumbar burst fractures can be associated with lamina fractures and can have dural tears and/or entrapped nerve roots.^26^ Laminectomy and reduction of the displaced roots, along with dural repair, in the neurologically intact patient, is still considered controversial. Nonetheless, we have found that using careful microdissection techniques, this approach is both safe and efficacious. Exploration and repair should be taken up in patients with a proven neurological deficit.

## CONSERVATIVE TREATMENT

Conservative treatment has a limited role in established cases with neurological deficit and instability. In cases without neurological deficit and instability, nonoperative treatment may play a role. However, this treatment is associated with increased risk for complications such as decubitus ulcers, deep vein thrombosis and pneumonia^6^ due to prolonged recumbency.

## STABILIZATION AND FUSION

Instability in thoracolumbar and lumbar burst fractures is usually caused by PLC disruption. The role of instrumentation is to restore immediate stability and correct the deformity. But in the long term, solid fusion has to be achieved to prevent the failure of the instrumentation. Without solid fusion, the implants will eventually fail due to instrumentation fatigue failure which occurs from cyclic loading. Successful fusion requires a bone graft or bone graft substitute that has some essential characteristics such as osteogenicity (usually provided by bone cells), osteoinducitivity (the ability to activate and sustain the cascade of biochemical processes that lead to bony healing) and osteoconductivity (the ability to provide a scaffold to which the new bone can attach to and propagate).^27^

## ANTERIOR STABILIZATION AND FUSION

In the initial period of anterior instrumentation, adaptations of Harrington rod devices were used. However, with the development of the Kaneda instrumentation, a major step forward was taken for the treatment of thoracolumbar burst fractures. The basic instrumentation has two screws placed through a staple into the intact suprajacent and infrajacent vertebral bodies which are then connected to two cross-linked rods. The major advantage of the system is the ability to use these screws as anchors for distracting the corpectomy site to allow better strut graft placement^14^ as well as superior rigidity compared to the plate systems. In addition, sagittal plane correction of the kyphosis which typically is seen with burst fractures is able to be best achieved by placement of a strut into the anterior column. Kaneda devices have about twice the stiffness of posterior constructs with axial compressive and torsional loading.^28^ However, the scenario has changed over a period of time due to innovative designs and techniques of the newer plate-screw systems and they appear to be as or more stable than some of the dual-rod screw instrumentations.^29^ Other advantages of anterior stabilization include its ability to limit fusion to the level above and below the injured site.

Following corpectomy, the site must be filled with a cage and/or bone graft which can sustain axial compressive loads and maintain kyphotic correction. Bone graft choices include autograft (usually bone from the corpectomy or the iliac crest), allograft or cages filled with morselized autograft or allograft. We prefer to use autologous graft due to its osteogenic, osteoinductive and osteoconductive properties. Any kyphosis, if present, must be maximally corrected prior to placement of the cage. Slight distraction of the corpectomy site using the screw-staple anchor or a Kaneda-type device can facilitate this correction.

The graft should be placed as close to the anterior vertebral body as possible. Optimally, it should be centered along the endplates to ensure even distribution of the compressive loads. Axial compression can then be delivered using the vertebral body screws to ensure a tight interference fit between the endplates and the cage or the bone graft. This will reduce the graft dislodgement.

Immediate stability can be maximized with bicortical screw purchase. After placing the strut, it is important to neutralize any break in the operating table before securing the rods as this will avoid a coronal plane deformity. Cross-connectors play an important role in dual rod-screw systems as they improve the resistance to rotational, torsional and bending forces. One of the major disadvantages of the anterior approach is the increased morbidity and it must be weighed against its advantages.

## POSTERIOR STABILIZATION AND FUSION

Lately, pedicle screws have largely replaced the hooks and wires for posterior stabilization due to the biomechanical advantages, particularly in the thoracolumbar and lumbar regions. Pedicle screws provide three-column fixation as well.^14^ Another advantage of pedicle screws is their ability to restore stability with fewer anchoring points which can spare motion segments.

Although some studies have suggested short-segment fixation, this strategy may result in high rates of construct failure in many cases.^30^,^31^ In our practice we commonly instrument two levels above and below the injured segment for highly unstable fractures. However, cases with less severe instability can be managed with only one level above and below. Short-segment pedicle screw stabilization can be combined with anterior instrumentation as well.^30^ However, short-segment fixation is more durable in the low lumbar spine primarily due to larger pedicle sizes and anatomical lordotic alignment. Dickman *et al.* suggested that to maintain this lordotic curve, the rods must be contoured to avoid the sequelae of flat-back syndrome.^32^*In situ* bending has been found to weaken the screw-bone interface and is not advised.^30^

Pedicle screw placement is technically demanding and carries the potential risk for nerve root, spinal cord or vascular injury if the cortical borders are breached.^33^ A careful planning and intraoperative imaging are of enormous help in ensuring that screws are placed correctly. Screw breakage may occur more frequently with smaller diameter screws compared to larger ones.^32^ Ultimately, the size of the screw is determined by measuring the maximal transverse pedicle diameter on preoperative CT or MR images.

Fusion is facilitated by decorticating all exposed bony elements provided they are present. Interspinous ligaments should be resected to facilitate fusion between these bony surfaces. We recommend that large amounts of autologous bone graft, harvested from the iliac crest, should be placed over the exposed surfaces. Use of allograft bone should be reserved for cases where autologous bone is not available due to various medical reasons. On the other hand, allograft bone, demineralized bone matrix (DBM) and recombinant growth factors can be used alone as an alternative or in combination with autograft. However, none of these substitutes provide all three basic properties of autograft bone. Bone morphogenetic proteins (BMP) have excellent osteoinductive activity as noted in both preclinical animal studies and in human trials.^34^ However, some authors have suggested that large doses of BMP have been required to induce adequate bone formation in humans.^35^ Another concern with BMP is that a single dose of the recombinant protein may not be sufficient for an adequate osteoinductive response, especially in cases where there is compromised bone stock and vascularity.^36^ The cost of BMP is also very high. Probably the biggest concern for BMP is the excessive and uncontrolled bone growth. Lately there has been a lot of interest in the use of bone marrow as an adjunct to the spinal fusion procedure along with autograft or allograft. In 1998, Connolly reported 80% healing rate for numerous skeletal healing problems using marrow grafting.^37^

Recently, the use of osteoblast progenitor cells separated from the patient's bone marrow has shown promising results. The technique provides a less invasive method to augment local bone graft, allograft and osteoblast progenitor cells at the fusion site to achieve successful fusion.

It is our practice to prefer the posterior approach for patients with ASIA Class A injuries. This avoids the additional morbidity of the anterior approach. Resection of one or more pedicles may help to facilitate the decompression as well.

Loss of correction is one of the most common complications of posterior stabilization of thoracolumbar burst fractures. In these cases, fractures tend to collapse leading to kyphosis. We prefer anterior fixation for such cases. In cases where only posterior instrumentation is undertaken, an additional level above and below can be included to resist the forces which favor kyphosis.

## ANTERIOR-POSTERIOR (AP) STABILIZATION AND FUSION

One of the most compelling indications for AP stabilization and fusion is the very unstable fracture or fractures/subluxation with intact neurological status or incomplete spinal cord injury. It is our practice to undertake both the surgeries on the same day to expedite rehabilitation and recovery. However, the primary deciding factor for this is the medical condition and age of the patient. Anterior-posterior surgery increases the physiologic demands on the already compromised patient due to the increased blood loss and operative time. The benefits of the combined AP surgery offset the risks by adequate decompression, stabilization and fusion in a patient with a highly unstable spine injury and intact neurological function. The surgery itself is as described in the separate anterior and posterior stabilization sections.

## POSTOPERATIVE CONSIDERATIONS

An immediate neurological evaluation should be done upon arrival in the recovery room and portable supine AP and lateral radiographs should be obtained. Thromboembolic stockings and sequential compression devices should be continued throughout the recovery period. If evidence of a deep vein thrombosis or pulmonary embolus is detected, placement of a Greenfield filter is preferable to systemic anticoagulation in the early postoperative period. Suction drains are usually removed on the third postoperative day. Our practice is to obtain a CT scan for a more detailed assessment of the location of all spinal implants and to assess the adequacy of the bony decompression. Non-steroidal antiinflammatory drugs should be avoided in the postoperative period. Glassman *et al.*, reported adverse affects on spinal fusion with the use of these agents.^38^ Physical therapy should be started as soon as possible.

## CONCLUSION

The most common site of injury to the spine is the thoracolumbar junction which is the mechanical transition junction between the rigid thoracic and the more flexible lumbar spine. The most widely accepted form of instability has been proposed by Denis.^10^ He proposed that injury to the middle column i.e. the posterior portion of the vertebral body, posterior longitudinal ligament and posterior disc was sufficient to create instability. He also classified unstable fractures into three types: mechanical (1^st^ degree), neurological (2^nd^ degree) or combined mechanical/neurological (3^rd^ degree). A disrupted PLC usually leads to mechanically unstable burst fracture. Cases with no neurological deficit can often be managed conservatively. Nonoperative treatment options have a limited role in patients with neurological deficits. It is important to follow an algorithmic approach in the initial patient assessment, radiological workup and ultimate decision-making for management. Selection of an appropriate surgical management and approach requires an in-depth analysis of the different available methods of decompression, stabilization and fusion. We usually perform posterior surgery for cases with ASIA Class A type of SCI. The extent of instrumentation is usually two or three levels above and two levels below. For cases with incomplete injury with significant ventral bony compression (50% or more on axial CT scan), no motor deficit with only bowel and bladder dysfunction or significant kyphotic deformity, we prefer anterior surgery. However, in the majority of cases, either an anterior or a posterior approach is reasonable.

The choice of approach primarily depends on the operating surgeon and his/her results. The optimal timing of the decompression is critical. A progressive neurological deficit is one of the indications for immediate surgery to treat a thoracolumbar or lumbar burst fracture. While anterior surgery has the advantage of achieving excellent canal decompression and the benefit of short segment fusion, this is achieved at the cost of increased approach-related morbidity. Posterior surgery has the advantage of being an effective approach associated with less morbidity. At the same time, it lacks the benefit of short segment stabilization and fusion. Other limitations of this approach are the possibility of achieving inadequate decompression or construct failure leading to recurrence of deformity. Anterior-posterior stabilization and fusion should be undertaken for very unstable fracture or fractures/subluxation with intact neurological status or incomplete spinal cord injury. Finally, the decision regarding the type and approach of the surgery should be individualized based on the type of the injury and medical condition of the patient.
